# 1-(2,3-Dibenzimidazol-2-ylpropyl)-2-methoxybenzene Is a Syk Inhibitor with Anti-Inflammatory Properties

**DOI:** 10.3390/molecules21040508

**Published:** 2016-04-18

**Authors:** Eunji Kim, Young-Jin Son, Yanyan Yang, Ting Shen, Ikyon Kim, Adithan Aravinthan, Jong-Hoon Kim, Jae Youl Cho

**Affiliations:** 1 Department of Genetic Engineering, Sungkyunkwan University, Suwon 16419, Korea; im144069@gmail.com (E.K.); orchid1201@hotmail.com (Y.Y.); 2 Department of Pharmacy, Sunchon National University, Suncheon 57922, Korea; sony@sunchon.ac.kr; 3 Medical College, Qingdao University, Qingdao 266071, China; 4 Jiangsu Collaborative Innovation Center of Regional Modern Agriculture & Environmental Protection/Jiangsu Key Laboratory for Eco-Agricultural Biotechnology around Hongze Lake, Huaiyin Normal University, Huaian 223300, China; shenting198612@hotmail.com; 5 College of Pharmacy and Yonsei Institute of Pharmaceutical Sciences, Yonsei University, Incheon 21983, Korea; ikyonkim@yonsei.ac.kr; 6 Department of Physiology, College of Veterinary Medicine, Chonbuk National University, Iksan 54596, Korea; aravinthchandra@gmail.com

**Keywords:** 1-(2,3-dibenzimidazol-2-ylpropyl)-2-methoxybenzene, inflammatory responses, NF-κB, Syk

## Abstract

Inflammation is the protective action of our bodies against external pathogens by recognition of pathogen-associated molecular patterns (PAMPs) via pattern recognition receptors (PRRs). Proper regulation of inflammatory responses is required to maintain our body’s homeostasis, as well as there are demands to develop proper acute or chronic inflammation. In this study, we elucidated the regulatory mechanism of NF-κB-mediated inflammatory responses by a novel compound, 1-(2,3-dibenzimidazol-2-ylpropyl)-2-methoxybenzene (DBMB). We found that DBMB suppressed inflammatory mediators, nitric oxide (NO) and prostaglandin E_2_ (PGE_2_), reacted to exposure to a number of toll like receptor (TLR) ligands. Such observations occurred following to decreased mRNA expression of several pro-inflammatory mediators, and such diminished mRNA levels were caused by inhibited transcriptional factor nuclear factor (NF)-κB, as evaluated by luciferase reporter assay and molecular biological approaches. To find the potential targets of DBMB, we screened phosphorylated forms of NF-κB signal molecules: inhibitor of κBα (IκBα), IκB kinase (IKK)α/β, Akt, 3-phosphoinositide dependent protein kinase-1 (PDK1), p85, and spleen tyrosine kinase (Syk). We found that DBMB treatment could suppress signal transduction through these molecules. Additionally, we conducted *in vitro* kinase assays using immunoprecipitated Syk and its substrate, p85. Consequently, we could say that DBMB clearly suppressed the kinase activity of Syk kinase activity. Together, our results demonstrate that synthetic DBMB has an effect on the inflammatory NF-κB signaling pathway and suggest the potential for clinical use in the treatment of inflammatory diseases.

## 1. Introduction

Inflammation is the biological response to protect our bodies from infection of pathogens such as bacteria, fungi, and viruses [1]. Inflammatory responses are initiated by recognition of pathogen-associated molecular patterns (PAMPs), which are recognized by PRRs. Four PRR families have been classified, and one of these is toll-like receptors (TLRs) [2]. TLRs recognize different types of ligands and the engagement of TLRs activates several inflammatory signaling pathways, for example, the activator protein (AP)-1 signaling pathway or the nuclear factor (NF)-κB signaling pathway [3,4]. By activating inflammatory signaling cascades, various pro-inflammatory cytokines and mediators are up-regulated, and released to regulate inflammatory responses and remove pathogens [5,6,7]. These reactions are mediated by various innate immune cells, the major being macrophages. The activation of macrophages relied upon interaction between the ligand and the TLR, and the consequent TLR signaling [6,8].

**Figure 1:**
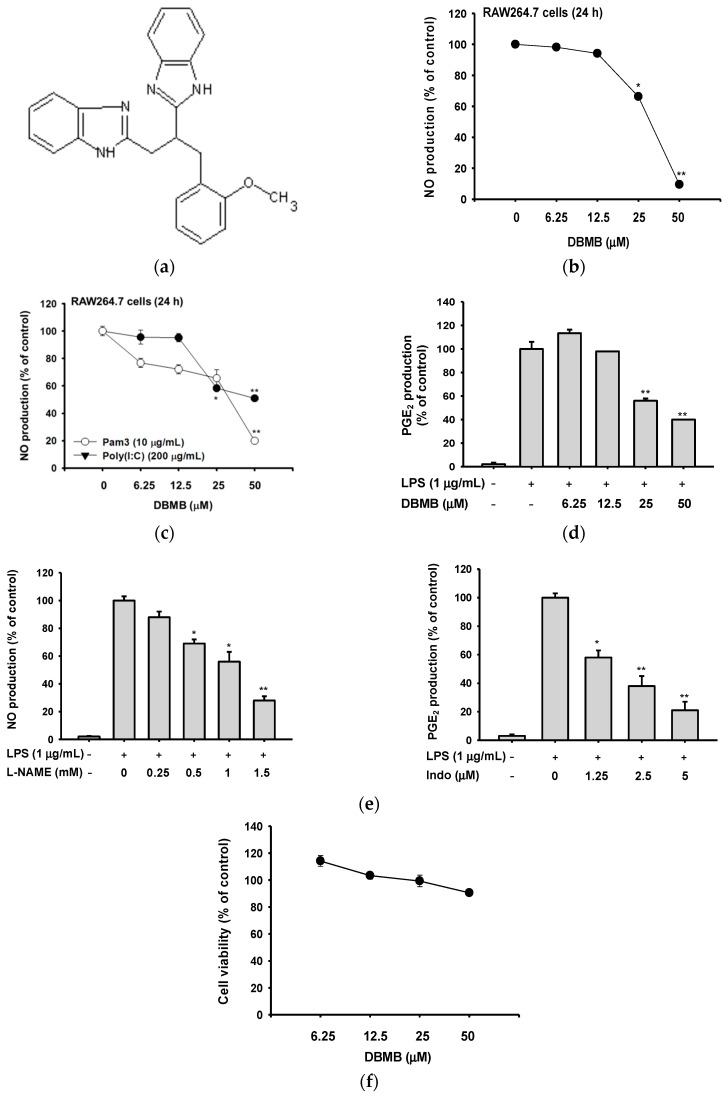
The structure of DBMB, and its effect on production of inflammatory mediators and cell viability. (**a**) The chemical structure of DBMB. (**b**, **c**, and **e** left panel) The NO production level was determined by Griess assay using RAW264.7 cells culture supernatants treated with DBMB or l-NAME and immune stimulant molecules. (**d** and **e** right panel) The level of PGE_2_ production was analyzed by ELISA using cell culture supernatants of RAW264.7 cells treated with DBMB or Indo (indomethacin) and LPS (1 μg/mL). (**f**) Cell cytotoxicity of DBMB was tested by MTT assay. All values (**b**–**f**) are presented as means ± standard deviations (SDs). * *p* < 0.05 and ** *p* < 0.01 compared with controls.

The NF-κB signaling pathway could be induced by TLR4, with the upstream molecules, spleen tyrosine kinase (Syk) and Src tyrosine kinases, activated sequentially [9,10]. As a result of Syk and Src activation, NF-κB signaling molecules, including phosphoinositide-3-kinase (PI3K)/p85, phosphoinositide-dependent kinase-1 (PDK1), protein kinase B (Akt), IκB kinase (IKK) α/β and IκBα are phosphorylated, and phosphorylation of those molecules is required to translocate transcriptional factors to the nucleus [11,12,13,14]. Translocation of NF-κB (p65/p50) is responsible for the transcription of pro-inflammatory genes involved in producing cytokines, mediators, and enzymes such as inducible nitric oxide (NO) synthase (iNOS), cyclooxygenase-2 (COX-2), and tumor necrosis factor (TNF)-α [15,16,17]. In this study, we investigated whether 1-(2,3-dibenzimidazol-2-ylpropyl)-2-methoxybenzene (DBMB, Figure 1a) regulates the expression of inflammatory mediators in lipopolysaccaride (LPS)-treated macrophages. In addition, we determined which signaling molecules were inhibited by DBMB in the NF-κB signaling pathway.

## 2. Results

### 2.1. DBMB Suppressed the Production of Pro-Inflammatory Mediators

We tested the inhibitory activity of DBMB on the production of pro-inflammatory molecules after treatment with several TLR ligands. When unprimed RAW264.7 cells were treated with LPS, Pam3CSK4, and Poly I:C, NO level in culture supernatants was dramatically enhanced from 0.72 μM to 54.7, 22.8, and 30.1 μM, respectively. Under these conditions, it was found that DBMB is able to dose-dependently suppress NO production triggered by the TLR ligands (Figure 1b,c). The upregulated release (56.77 ng/mL) of prostaglandin E_2_ (PGE_2_) by LPS from unprimed cells (1.08 ng/mL) was also decreased up to 60% by DBMB under LPS-treated conditions (Figure 1d). Two standard compounds, l-*N*^G^-nitroarginine methyl ester (l-NAME) and indomethacin (Indo), showed dose-dependent inhibitory patterns on NO and PGE_2_ production (Figure 1e). Cell viability was evaluated by the (3-4,5-dimethylthiazol-2-yl)-2,5-diphenyltetrazolium bromide (MTT) assay after treatment of DBMB for 24 h. There was no cytotoxic effect on RAW264.7 cells, with DBMB up to 50 μM (Figure 1f), implying that all pharmacological data observed with DBMB (0 to 50 μM) in this study are not derived by drug’s nonspecific activity. These results support that the anti-inflammatory effect of DBMB, and show that it was not caused by any cytotoxicity of DBMB.

### 2.2. DBMB Inhibited Inflammatory Responses at the Transcriptional Level

**Figure 2:**
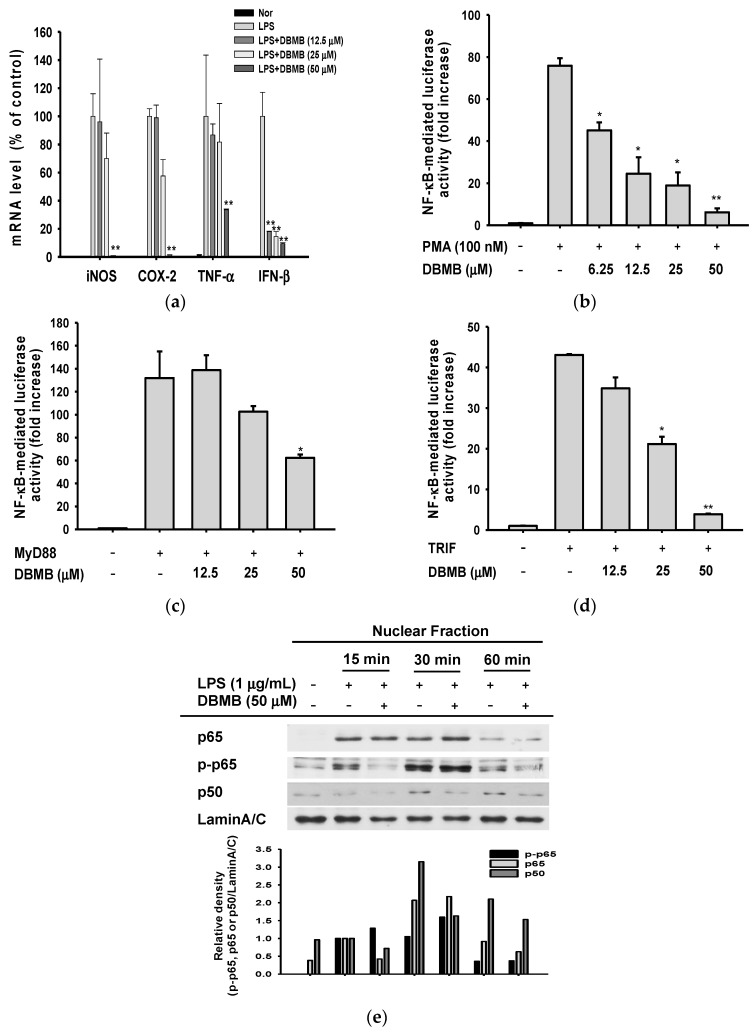
Effect of DBMB on mRNA expression level of inflammatory mediators, and the transcriptional activation of NF-κB. (**a**) mRNA expression levels of pro-inflammatory mediators in RAW264.7 cells treated with DBMB and LPS (1 μg/mL); (**b**) NF-κB promoter activity was tested by luciferase assay under PMA (100 nM) treatment condition with DBMB on NF-κB reporter gene and β-galactosidase gene (as a control) transfected HEK293 cells; (**c**,**d**) Luciferase reporter activity was determined by NF-κB luciferase reporter gene assay, in FLAG-MyD88 or CFP-TRIF plasmid transfected HEK293 cells; (**e**) The translocation level of NF-κB transcription factor subunits, p65 and p50, was determined by immunoblotting with antibodies against phospho- or total proteins in the nuclear fraction of LPS-treated RAW264.7 cells. All values (**a**–**d**) are presented as means ± SDs. * *p* < 0.05 and ** *p* < 0.01 compared with controls.

To investigate whether the anti-inflammatory effects of DBMB were regulated at the transcriptional level, we prepared mRNA from LPS-treated RAW264.7 cells with DBMB. Real-time polymerase chain reaction (PCR) was performed to verify the expression levels of pro-inflammatory cytokines and molecules (Figure 2a) [18,19,20]. DBMB significantly suppressed the mRNA expression of inflammatory enzymes iNOS and COX-2. mRNA expression of TNF-α and IFN-β were also decreased by DBMB in LPS induction condition.

To identify whether decreased mRNA expression levels of pro-inflammatory genes resulted from transcriptional factor suppression, we performed promoter assays using an NF-κB promoter luciferase construct. Phorbol 12-myristate 13-acetate (PMA) has been known as a protein kinase C (PKC) activator, and PKC is implicated in NF-κB activation [21,22]. We transfected a NF-κB promoter luciferase gene and β-galactosidase construct into HEK293 cells, and activated the NF-κB signal cascade by PMA treatment (Figure 2b). We found that NF-κB-mediated luciferase activity was dose-dependently decreased by DBMB. Next, we co-transfected a TLR4 adaptor molecule constructs, expressing myeloid differentiation primary response gene 88 (MyD88) and toll/interleukin-1 receptor-domain-containing adapter-inducing interferon-β (TRIF), and an NF-κB promoter luciferase construct, to HEK293 cells. In each, the activity of NF-κB transcriptional factor during DBMB exposure for 24 h, was examined. Figure 2c,d shows that DBMB reduced the promoter binding activity of NF-κB. Continuously, we prepared nuclear fraction from LPS-stimulated RAW264.7 cells with DBMB to ascertain the translocation of NF-κB subunits, p65 and p50, into the nucleus (Figure 2e). Phosphorylated p65 and p50 (active forms to transcribe several inflammatory genes [23,24]), were decreased at 15 min, and the nuclear level of total p50 was reduced at 15, 30, and 60 min, consequentially. These results imply that DBMB could affect the activation of NF-κB.

### 2.3. The NF-κB Signaling Cascade Was Modulated by DBMB

**Figure 3:**
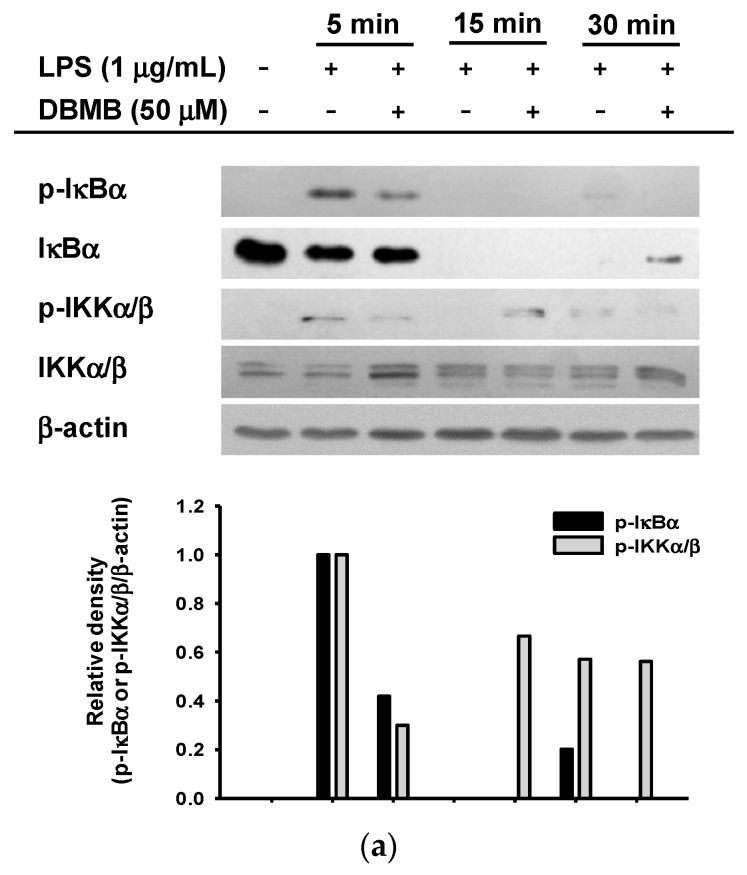

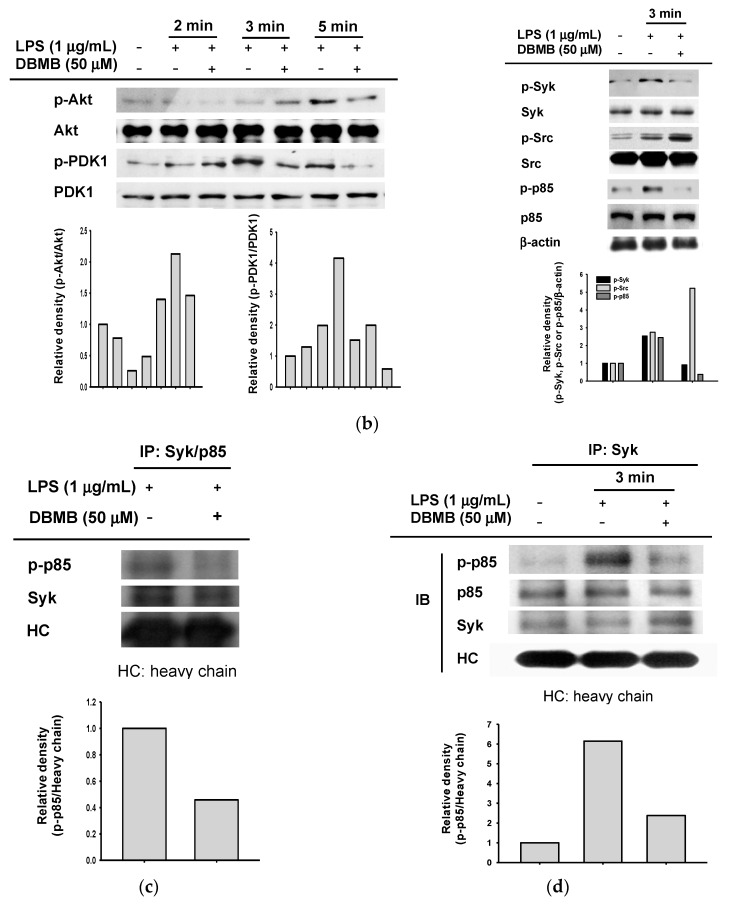
The effect of DBMB on NF-κB signaling pathway regulation. (**a**,**b**) Phosphorylation level of NF-κB signaling molecules as determined by immunoblotting using IκBα, IKKα/β, Akt, PDK1, p85, Syk, and Src antibodies against phosphor- or total protein in whole RAW264.7 cells lysates; (**c**) Direct Syk kinase activity was determined by immunoblotting using immunoprecipitated p85 and Syk with ATP. Phospho-p85 and total Syk antibodies were used; (**d**) Suppression of Syk kinase activity was determined by immunoprecipitation using total level of Syk antibody. Phospho- or total proteins of Syk and p85 in immunoprecipitation mixture were determined by immunoblotting using antibodies against phospho- and total Syk and p85. Please explain the meaning of “+” and “−” here.

We next examined the NF-κB signaling pathway using whole cell lysate of LPS treated RAW264.7 cells, with or without DBMB. The NF-κB pathway is controlled by signals generated by kinase phosphorylation [23]. First, we checked phosphorylation levels of IκBα and IKKα/β by immunoblot analysis.DBMB blocked phosphorylation of IκBα and IKKα/β following activation of LPS (Figure 3a). Following this, we measured levels of phosphorylated Akt, PDK1, p85, Syk, and Src, which are upstream molecules in the NF-κB pathway [25,26]. DBMB inhibited the phosphorylation of Akt at 5 min and PDK1 at 4 and 5 min (Figure 3b left panel). The tyrosine kinases, Syk and Src, are known to initiate p85/PI3K activation, which is critically important in NF-κB pathway [27]. The phosphorylation of Syk and p85 was blocked at 3 min, while there was no inhibitory activity of DBMB on Src kinase (Figure 3b right panel). Based on these finding, we suppose that Syk kinase might be a direct target of DBMB.

We then conducted a Syk kinase assay to confirm whether the inhibitory target of DBMB is Syk. In order to do this, we immunoprecipitated Syk from RAW264.7 cells induced by LPS for 10 min and immunoprecipiated p85/PI3K, a downstream molecule phosphorylated by Syk [28,29,30], from unstimulated RAW264.7 cells; then these Syk and p85 were mixed with 200 μM ATP. We found phosphorylation level of p85 was decreased when DBMB was treated (Figure 3c), confirming that DBMB is able to block phosphorylation capacity of Syk kinase to its substrate. We further tested the interaction of Syk and its substrate p85 by immunoprecipitation (Figure 3d). In agreement with previous data, phospho-Syk was strongly inhibited at 3 min. In agreement with our Syk kinase assay results, phospho-p85 was considerably abrogated. These results imply that DBMB has an ability to suppress Syk kinase activity.

## 3. Discussion

In this study, we have evaluated the anti-inflammatory action of DBMB, which is functionally unknown yet, to develop novel anti-inflammatory drug. We have shown that DBMB suppressed the secretion of NO and PGE_2_ under LPS-treated conditions (Figure 1a,b), and NO production is reduced by DBMB upon treatment with the TLR2 ligand, Pam3CSK4, and the TLR3 ligand, Poly (I:C) (Figure 1c). Together, cytokines and mediators were reduced following the down-regulation of transcriptional activation level, as determined by real-time PCR and NF-κB reporter gene assay (Figure 2a–d). This indicates that DBMB may be capable of negatively regulating inflammatory responses produced from macrophages exposed to various inflammatory stimuli.

NF-κB is composed of five subunits, c-Rel, Rel A (p65), Rel B, p50, and p52, and they form various homo- or heterodimers that enter the nucleus. As a transcriptional factor, NF-κB should be released from IκBα and translocated into the nucleus in a phosphorylated form. Translocation of dimers occurs through post-translational modification including ubiquitination of IκBα and phosphorylation of NF-κB subunits by different stimuli such as LPS or TNF-α [31,32]. Classically, proteolysis of IκBα arises from phosphorylation by IκB kinase (IKK), consisting of IKKα and IKKβ. [31,33]. As we expected, DBMB inhibited the translocation of NF-κB subunits, p65 and p50, when LPS-activated inflammatory conditions (Figure 2e). The levels of phosphorylated proteins (IκBα and IKKα/β) in the whole cell lysate were also decreased in DBMB-mediated anti-inflammatory action (Figure 3a).

Moreover, the transcriptional factor NF-κB is reported to be involved in important physiological processes including inflammation and apoptosis, and is also known to control gene expression related to these responses [13,34,35]. During an inflammatory response, NF-κB modulates the transcription of pro-inflammatory genes such as TNF-α, iNOS, COX-2, chemokines, and adhesion molecules [24,36,37]. In our study, we limited testing to the expression level of only a few cytokines, but we could expect that DBMB has the capability of regulating, not only diverse chemokines, but also adhesion molecules managed by NF-κB activity. Decreased cell adhesion molecule expression is also known as a way to lower inflammatory responses [1,38]. To ensure the suppressive effect of DBMB in inflammatory responses is valid, we will aim to determine the expression pattern of adhesion molecules.

When macrophages are exposed to LPS, upstream elements of the NF-κB pathway including Syk and Src kinases, are activated. Indeed, numerous reports have suggested a possibility that Syk and Src kinases can be targeted by anti-inflammatory strategies [12,30,39,40]. Interestingly, our immunoblot analysis implied that DBMB could target Syk kinase activity in its anti-inflammatory action (Figure 3b). In addition, the inhibitory activity of DBMB on Syk kinase was also confirmed by molecular biological approaches and kinase assay (Figure 3c,d). In agreement with these results, downstream proteins (e.g., p85/PI3K) were inactivated and subsequent inhibition of transcriptional activation was observed. In spite of this, the mechanism by which DBMB directly suppresses Syk kinase activity is not yet fully understood. Most Syk inhibitors are known to act as ATP-competitive inhibitors, and these inhibitors have the potential to be used clinically to treat allergy or autoimmune diseases. Many reports have delineated that Syk kinase has a pivotal role in immunological functions [26,30,41,42]. To advance our understanding, further study will focus upon the inhibitory action upon Syk kinase activity and the additional immunoregulatory activity of DBMB.

## 4. Materials and Methods

### 4.1. Materials

1-(2,3-Dibenzimidazol-2-ylpropyl)-2-methoxybenzene (DBMB, ST005141, C_24_H_22_N_4_O, M.W.: 382.46, and Purity: 97%) was purchased from ActiMol Organic Compound Collections (Newark, DE, USA). l-NAME, indomethacin, MTT, and lipopolysaccharide (LPS, E. coli 0111:B4) were obtained from Sigma Chemical Co. (St. Louis, MO, USA). A luciferase construct containing binding promoters for NF-κB was used as reported previously [43]. DNA constructs with the FLAG-MyD88 and CFP-TRIF genes were used as reported previously [44,45,46]. Fetal bovine serum (FBS), RPMI1640 and DMEM were purchased from Gibco BRL (Grand Island, NY, USA). RAW264.7 cells, a BALB/c-derived murine macrophage cell line (ATCC No.: TIB-71), and HEK293 cells, which are a human embryonic kidney cell line (ATCC No.: CRL-1573), were obtained from ATCC (Rockville, MD, USA). All other chemicals were purchased from Sigma. Phospho-specific and total antibodies against p65, p50, Akt, p85, PDK1, Src, Syk, IKKα/β, IκBα, lamin A/C, and β-actin were purchased from Cell Signaling (Beverly, MA, USA).

### 4.2. Cell Culture

RAW264.7 and HEK293 cells were cultured in RPMI 1640 and DMEM medium, respectively, each supplemented with 10% heat-inactivated FBS and antibiotics (penicillin and streptomycin) at 37 °C in 5% CO_2_. For each experiment, RAW264.7 cells were detached using a cell scraper, and HEK293 cells were trypsinized. At the cell density used in our experiments (2 × 10^6^ cells/mL), the proportion of live cells was more than 99%, when measured by Trypan blue dye exclusion tests.

### 4.3. NO and PGE_2_ Production

Following pre-incubation of RAW264.7 cells (1 × 10^6^ cells/mL) overnight, cells were pre-treated with DBMB (0 to 50 μM) for 30 min and further cultured with LPS (1 μg/mL) for 24 h. The inhibitory effect of DBMB on NO and PGE_2_ production was determined by analyzing NO using a Griess assay and an ELISA, respectively, as previously described [47,48].

### 4.4. Cell Viability Test

Following pre-incubation of RAW264.7 cells (1 × 10^6^ cells/mL) overnight, cells were pre-treated with DMBM (0 to 50 μM) and incubated for 24 h. The cytotoxic effect of DBMB was then investigated using a conventional MTT assay, as previously described [49]. Three hours prior to culture termination, 10 μL of MTT solution (10 mg/mL in phosphate-buffered saline (PBS) pH 7.4) was added to the cultures, and the cells were continuously incubated until the termination of this test. The incubation was finalized by treating 15% sodium dodecyl sulfate to each well, to solubilize the formazan [50]. The absorbance at 570 nm (OD570-630) was measured using a Spectramax 250 microplate reader (Molecular Devices, Sunnyvale, CA, USA).

### 4.5. mRNA Analysis by Quantitative Real-Time PCR

To examine cytokine mRNA expression levels, RAW264.7 cells pre-treated with DBMB (0 to 50 μM) for 30 min were incubated with LPS (1 μg/mL) for 6 h. Total RNA from DBMB-treated cells was then isolated with TRIzol Reagent (Gibco BRL), as mentioned by the manufacturer. The total RNA was stored at −70 °C for future use. Semi-quantitative RT reactions were carried out as instructed before [51]. The mRNA quantification was performed by real-time PCR with SYBR Premix Ex Taq, as the manufacturer instructed (Takara Bio, Inc., Shiga, Japan) using RT-thermal cycler (Bio-Rad, Hercules, CA, USA), as described in previous papers . The results were expressed as a ratio of the optimal density relative to GAPDH. The primers used (Bioneer, Seoul, Korea) are listed in Table 1.

### 4.6. Preparation of Total Lysates, Nuclear Extracts, and Immunoblotting

The RAW264.7 cells (5 × 10^6^ cells/mL) were washed in cold PBS with 1 mM sodium orthovanadate and lysed using a sonicator (Thermo Fisher Scientific, Waltham, MA, USA) in lysis buffer for 30 min with rotation at 4 °C [43,52]. The lysates were clarified by centrifugation at 16,000× *g* for 10 min at 4 °C and stored at −20 °C until before the next experiments. The cell nuclear lysates were prepared in a three-step procedure, according to previous method [53]. Following this treatment, the cells were harvested with a rubber policeman and washed with 1 × PBS. The cells were then lysed in 500 μL lysis buffers on ice for 4 min. Cell lysates were then spin-downed at 12,000 rpm for 1 min in a microcentrifuge. In the second step, the pellet (the nuclear fraction) was washed with the lysis buffer without Nonidet P-40. In the final step, the nuclei were treated with an extraction buffer (lysis buffer containing 500 mM KCl and 10% glycerol). The nuclei/extraction buffer mixture was kept to freeze at −80 °C and then thawed on ice and spin-downed at 19,300× *g* for 5 min. The supernatant was collected as the nuclear extract. Soluble cell lysates (30 μg/lane) were subjected to immunoblotting. The total levels of transcription factors (p65 and p50), Akt, PDK1, p85, IκBα, IKKα/β, Syk, Src, Lamin A/C, and β-actin were visualized as described previously [54]. The relative intensity of phospho-proteins was calculated using total-protein levels with the DNR Bio-imaging system (Jerusalem, Israel).

### 4.7. Transfection of DNA and Luciferase Reporter Gene Activity Assay

HEK293 cells were transfected with empty vector or the indicated plasmids (FLAG-MyD88 and CFP-TRIF) (0.3 μg/mL), NF-κB-Luc (0.3 μg/mL) and β-galactosidase (0.1 μg/mL) using polyethylenimine (PEI) in a 24-well plate as reported previously [55]. Following 24 h, the transfected cells were treated with DBMB for an additional 24 h. The cells were finally harvested and lysed to evaluate luciferase activity. The luciferase assays were carried out with the luciferase assay system (Promega, Madison, WI, USA), as indicated previously [56]. The luciferase activity was normalized to β-galactosidase activity.

### 4.8. In Vitro Syk Kinase Assay Using Immunoprecipitated Proteins

For determining the usage of ATP by Syk kinase, Syk was immunoprecipitated from lysates of LPS-treated RAW264.7 cells for 10 min with an anti-Syk antibody. p85 was immunoprecipitated from untreated RAW264.7 cells using an anti-p85 antibody to serve as a kinase substrate. The kinase assay was carried out for 30 min at 30 °C in a 50 μL reaction volume including 200 μM ATP, as well as other components, from a kinase assay kit (Upstate Biotechnology, Lake Placid, NY, USA), as per the manufacturer's protocol and a previous report [57]. The incubation mixture was then additionally analyzed by immunoblotting to determine the kinase activity of immunoprecipitated Syk with anti-phospho-p85.

### 4.9. Immunoprecipitation

Cell lysates containing equal amounts of protein (500 μg) from RAW264.7 cells (1 × 10^7^ cells/mL) treated with or without LPS (1 μg/mL) for 3 min were prepared. Samples were incubated with 5 μL anti-Syk overnight at 4 °C. Immune complexes were mixed with 20 μL protein A-coupled Sepharose beads (50% *v*/*v*) and rotated for 4 h at 4 °C. Boiled immune complexes were then immunoblotted, and the levels of phosphorylated or total Syk and p85, were determined as reported previously [25,58].

### 4.10. Statistical Analysis

All values are presented as means ± standard deviations (SDs) of six technical replicates. For statistical comparisons, results were analyzed using ANOVA/Scheffe’s *post hoc* test and the Kruskal–Wallis/Mann–Whitney tests. A *p*-value < 0.05 was considered statistically significant. All statistical tests were carried out using SPSS software (SPSS Inc., Chicago, IL, USA). Similar results were obtained in a second biological replicate for all *in vitro* experiments.

## 5. Conclusions

**Figure 4:**
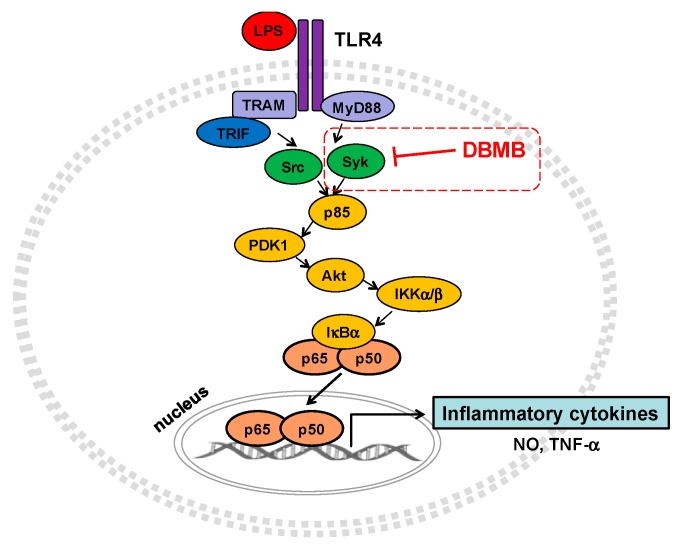
Putative inhibitory action of DBMB on the NF-κB pathway. Within the NF-κB pathway, DBMB inhibited Syk kinase activity, as well as sequentially suppressing the activities of down-stream kinases. As a result of Syk kinase inhibition, the expression of inflammatory cytokines was decreased, and DBMB shows anti-inflammatory activity.

In summary, in this study we have deciphered how DBMB suppressed inflammatory signal transduction following macrophages exposure to inflammatory stimulation, and found that Syk kinase maybe a direct target of DBMB (Figure 4). The targeting effect of DBMB on Syk kinase suggests the potential for this compound to be developed as a new drug against inflammatory diseases.

## Figures and Tables

**Table 1:** PCR primers used in this experiment.

Name (Real-time PCR)		Sequence (5′ to 3′)
iNOS	F	GGAGCCTTTAGACCTCAACAGA
	R	TGAACGAGGAGGGTGGTG
TNF-α	F	TGCCTATGTCTCAGCCTCTTC
	R	GAGGCCATTTGGGAACTTCT
COX-2	F	GGGAGTCTGGAACATTGTGAA
	R	GCACATTGTAAGTAGGTGGACTGT
IFN-β	F	TCCAAGAAAGGACGAACATTCG
	R	GAGGCCATTTGGGAACTTCT
GAPDH	F	CAATGAATACGGCTACAGCAAC
	R	AGGGAGATGCTCAGTGTTGG

## Abbreviations

The following abbreviations are used in this manuscript:

MTT	(3-4,5-dimethylthiazol-2-yl)-2,5-diphenyltetrazolium bromide
DBMB	1-(2,3-dibenzimidazol-2-ylpropyl)-2-methoxybenzene
COX-2	cyclooxygenase-2
FBS	fetal bovine serum
Indo	indomethacin
iNOS	inducible nitric oxide synthase
IKK	IκB kinase
LPS	lipopolysaccharide
PAMPs	pathogen-associated molecular patterns
PBS	phosphate-buffered saline
PRRs	pattern recognition receptors
PI3K	phosphoinositide-3-kinase
PDK1	phosphoinositide-dependent kinase-1
Akt	protein kinase B
PKC	protein kinase C
TLRs	toll-like receptors
TNF	tumor necrosis factor
